# The realisation of targeted antitumour therapy

**DOI:** 10.1038/sj.bjc.6602602

**Published:** 2005-05-31

**Authors:** R Bicknell

**Affiliations:** 1 Angiogenesis Laboratory, Cancer Research UK, Weatherall Institute of Molecular Medicine, University of Oxford, Oxford OX3 9DS, UK

**Keywords:** targeted therapy, antitumour, angiogenesis, VEGF, EGF

## Abstract

Better understanding of the pathways regulating proliferation and metastasis of cancer cells has led to the development of novel molecular-targeted therapies. The number of molecular-targeted agents approved for use in the clinic is growing, with many more in clinical trials. Most of these compounds can be broadly classified into two main categories: monoclonal antibodies and small-molecule tyrosine kinase inhibitors. The pathological processes targeted include vascular endothelial growth factor-dependent tumour angiogenesis and epidermal growth factor receptor-dependent tumour cell proliferation and survival. Unlike conventional chemotherapy, molecular-targeted agents offer the potential advantages of a relatively high therapeutic window and use in combination with other anticancer strategies without overlapping toxicity. It is hoped that these drugs will become valuable therapeutic tools within the multimodal approach to treating cancer. Recent progress in targeted antitumour therapy is discussed, with a focus on antiangiogenesis.

Front-line therapy for cancer not accessible to surgical intervention has for the last 30 years remained a combination of chemo- and radiotherapy. Despite recent advances in patient survival resulting from the development of newer generation cytotoxic agents such as the taxanes and gemcitabine, the overall prognosis for patients with most common cancers remains poor. Failure to improve conventional cytotoxic therapy over a period of several decades has led cancer researchers to search for completely novel approaches to treat cancer.

The present time is an exciting one for those involved in cancer research and treatment, as several of the strategies embarked upon a decade ago to enable targeting of tumour therapy are now achieving fruition (Green, 2004). Such approaches have evolved from a greater understanding of the pathways regulating cell proliferation and metastasis. These studies have identified novel cancer targets and enabled the subsequent development of targeted therapeutics. The number of new agents approved for use in the clinic is growing, as are the number of anticancer clinical trials. Of more significance is that the vast majority of the anticancer clinical trials now in progress involve novel drugs such as angiogenesis inhibitors (nearly one half) rather than chemically modified conventional cytotoxic agents. It is hoped that these novel agents will become valuable therapeutic tools used in combination with certain conventional cytotoxic agents within a multimodal approach to the treatment of cancer. It is anticipated that such treatments will lead to significant improvements in cancer therapy.

## LIMITATIONS OF CONVENTIONAL THERAPY

As noted in the introduction, the mainstay of cancer chemotherapy in recent decades has been the use of cytotoxic agents. In effect, one attempts to identify an agent that kills tumour in preference to normal cells. Despite expenditure of vast effort on the development of cytotoxic agents, progress in recent years has been slow. In particular, the narrow therapeutic index of cytotoxic drugs and the emergence of drug resistance mean an unfavourable prognosis for many cancer patients.

## ANGIOGENESIS AND TUMOUR DEVELOPMENT

The majority of human cancers arise from epithelial tissues in which the epithelial cells are undergoing continuous replication to maintain the protective layer of cells in contact with the environment. Examples include the lining of the lung and digestive tract and in the epithelium of secretory organs such as the breast and prostate. The malignant transformation of normal human epithelial cells into carcinoma cancer cells is a complex, multistep process. An essential change required for the development of all cancers is mutation in appropriate genes. There are usually several genetic changes involving, for example, proto-oncogenes, tumour-suppressor genes and genes that regulate cell cycle events. Once a cell has lost normal control of its growth and division, it expands and multiplies to form the initial primary tumour. At this stage, the tumour is fed by diffusion from nearby blood vessels; however, as the tumour cells increase in number they eventually become starved of nutrients such as oxygen and glucose. At this point during the early development of a tumour, the angiogenic switch occurs, and this is of particular relevance to therapeutic approaches that target angiogenesis (Bergers and Benjamin, 2003). The angiogenic switch occurs when the net balance of endogenous inhibitors and activators of angiogenesis within the microenvironment of the developing tumour shifts in favour of angiogenesis. While there is some evidence that this switch may involve an irreversible change in the developing carcinoma cell itself, it is also plausible that the cell experiences a stress response to the lack of oxygen (hypoxia) and other nutrients within the microenvironment of the developing tumour. For example, many normal cells will release vascular endothelial growth factor (VEGF) and other proangiogenic factors on exposure to hypoxia. Once a proangiogenic environment is achieved, new blood vessels grow into the tumour, sustaining tumour growth and development. In addition, the disorganised and immature features of the tumour neovasculature provide readily accessible exit points for haematogenous spread of cancer cells, a forerunner of disseminated disease and metastasis.

## INHIBITION OF TUMOUR ANGIOGENESIS

That tumours require new vasculature to grow and that inhibition of the growth of tumour vasculature or antiangiogenesis could be an effective anticancer strategy has been the subject of intense research. The idea had been around for some time, but was popularised in the 1990s by researchers such as Judah Folkman, who collected the evidence arguing that angiogenesis is needed for tumour growth in a cogent article entitled ‘What is the evidence that growth of solid tumours is angiogenesis dependent?’ (Folkman, 1990). An increase in angiogenesis research and several key advances rapidly followed. For example, it became routinely possible to culture endothelial cells *in vitro*, reliable and reproducible *in vivo* angiogenesis assays were developed and at the molecular level, potent peptide angiogenic factors such as fibroblast growth factor and VEGF were identified. Two key tenets supported early research into tumour antiangiogenesis. Firstly, as angiogenesis is largely absent in the healthy adult, it was widely held that an antiangiogenic approach would be without side effects, and secondly, the fact that the endothelial cells involved in angiogenesis are normal cells meant that they did not suffer the genome instability seen in carcinoma cells and as such were unlikely to develop drug resistance. Drug resistance had long proven a problem in targeting carcinoma cells directly.

Most deaths from cancer result from metastases and metastatic lesions are an important target of antiangiogenic therapy. The problem with metastases is the difficulty of treatment. Surgery is rarely an option once the tumour has spread beyond a limited area and the same applies to radiotherapy, hence the attraction of systemic therapy and the sustained efforts to develop it.

A large body of evidence has confirmed VEGF as a key regulator of tumour angiogenesis (Ferrara *et al*, 2003). Vascular endothelial growth factor is induced by both hypoxia and glucose deprivation. It is found to a greater or lesser extent in all solid tumours and is a potent angiogenic factor *in vivo*. The fact that VEGF was (until recently) thought to be a growth factor specific for endothelium and not widely expressed outside of tissue undergoing active angiogenesis (tumours, wounds, tissues that form part of the female reproductive tract) identified VEGF as a key molecular target for antiangiogenic therapy. It is now generally accepted that VEGF-induced angiogenesis and hyperpermeability are mediated via binding to the VEGF receptor-2 (VEGFR-2) located on the surface of vascular endothelial cells. Binding causes VEGFR-2 dimerisation and induces tyrosine kinase (TK)-mediated autophosphorylation, which in turn leads to activation of the relevant downstream signalling pathways.

As is frequently the case, antibodies were used initially to obtain proof that VEGF is a valid antitumour target. Thus, in 1993 Ferrara and co-workers showed that a blocking antibody to VEGF inhibited the growth of human tumour xenografts in athymic mice (Kim *et al*, 1993). This study encouraged the development of anti-VEGF compounds as anticancer agents, including a humanised anti-VEGF antibody (bevacizumab [Avastin™]) (Ferrara *et al*, 2004) as well as small-molecule inhibitors of VEGFR-2 TK activity (Drevs *et al*, 2003).

## KEY MOLECULAR TARGETS FOR NOVEL ANTITUMOUR AGENTS

Although angiogenesis is an area of intense research, and both VEGF and VEGFR-2 are key molecular targets, there are several other molecules not involved in angiogenesis that possess suitable characteristics for targeting by novel antitumour agents. These include the epidermal growth factor receptor (EGFR [HER-1]) and the related receptor HER-2, Bcr-Abl, c-Kit, platelet-derived growth factor receptor (PDGFR) and CD20. Most of the molecular-targeted agents currently approved or undergoing clinical evaluation can be broadly classified into two main categories: monoclonal antibodies or small molecule TK inhibitors (Table 1). Monoclonal antibody therapy neutralises the ligands or receptors that are overexpressed in particular cancers (Harris, 2004). Various transmembrane receptors with intrinsic TK activity have been identified as regulators of tumour or tumour vessel growth (Drevs *et al*, 2003). The ‘target’ for TK inhibitors is the ATP-binding site within the kinase domain of these receptors.

Molecular-targeted agents offer a spectrum of selectivity, from single-target monoclonal antibodies such as bevacizumab (Ferrara *et al*, 2004) to a TK inhibitor such as vatalanib, which may inhibit several targets (Wood *et al*, 2000). The selectivity profile of targeted agents is a two-edged sword. A single-target drug may be expected to have a better tolerability profile, thus increasing the likelihood for its use in combination. However, a precise mechanism of action means that the efficacy of single-target agents is more susceptible to the development of tumour resistance/redundancy. A more robust option for long-term treatment may be either a single molecule that ‘targets’ more than one pathway or a tailored combination of single-target drugs. An example of the former is ZD6474, which can inhibit two key pathways in tumour growth: VEGFR-dependent tumour angiogenesis and EGFR-dependent tumour cell proliferation and survival (Wedge *et al*, 2002).

Unlike conventional chemotherapy, targeted agents offer the potential advantages of a relatively high therapeutic window and use in combination with other anticancer strategies (chemotherapy, radiotherapy and other targeted agents) without significant overlapping toxicity.

## TRANSLATING THE PROMISE OF TARGETED THERAPY INTO CLINICAL BENEFIT

Ongoing use of approved agents, as well as further evaluation of new drugs, will reveal to what extent the clinical promise of targeted therapy is to be fulfilled. Most recently, the success with bevacizumab in advanced metastatic colorectal cancer was an encouraging demonstration of proof-of-principle of an anti-VEGF approach. Administration of bevacizumab with irinotecan, fluorouracil and leucovorin (IFL) gave a median survival of 20.3 months against 15.6 months for patients receiving IFL alone (Hurwitz *et al*, 2004). This result was a highly significant development not only in validating the VEGF signalling axis as a clinically valuable target in cancer therapy, but also in demonstrating that the clinical promise of targeted therapy can be translated into meaningful benefits for patients. Further results from clinical trials of bevacizumab and other molecular-targeted agents in earlier cancers are eagerly awaited. It could be that the optimal use of these agents will be in combination with other anticancer strategies, including other targeted therapies. For example, bevacizumab has shown its most dramatic clinical activity in combination with cytotoxic therapy, but is much less effective when administered alone (Hurwitz *et al*, 2004). This has led some to postulate that bevacizumab is effective not only in inhibiting tumour angiogenesis but also by enabling ‘normalisation’ of tumour vessels by removing excess VEGF produced by the tumour. Normalisation of tumour blood vessels by antiangiogenic agents may permit an increase in blood flow and consequently more efficient delivery of the cytotoxic agents to the tumour cells (Ellis, 2004; Jain, 2005). Clearly, much remains to be learned not only in terms of how antiangiogenic therapy exerts its antitumour properties but also in the design of optimal treatment schedules.

## CONCLUSIONS AND CONSIDERATIONS

Impressive as the clinical results with bevacizumab are it is starkly apparent that at best it inhibits but does not halt tumour growth, so there remains much to do. Why is the therapy not affecting more long-term survivors or even cures? The reasons are no doubt complex. One possibility is that VEGF is not the sole angiogenic factor produced by the tumour. Indeed, many publications have shown that most tumours express multiple angiogenic factors (Carmeliet and Jain, 2000; Kerbel and Folkman, 2002). Different stresses occurring in the tumour can induce (sometimes different) angiogenic factors. For example, hypoxia induces predominantly VEGF (Shweiki *et al*, 1992) whereas oxidative stress induces both VEGF and the strongly angiogenic interleukin-8 (Marjon *et al*, 2004). We have recently used a rat orthotopic xenograft bladder cancer model to show that while a blocking VEGF antibody reduced tumour take by 50%, tumour formation was abolished by concomitant administration in the drinking water of the oxidative stress blocker *N*-acetylcysteine (‘Parvolex’) (Brown *et al*, 2005).

A complementary anticancer strategy that also targets the endothelium is tumour vascular targeting (Neri and Bicknell, 2005). Destruction of the tumour vasculature, as opposed to the inhibition of new vessel growth, is an established idea that has recently attracted considerable renewed interest. The two approaches may be useful in combination, for example, destroy the tumour vasculature using a vascular targeting agent to elicit tumour collapse and then inhibit regrowth by long-term administration of an antiangiogenic agent.

The ultimate success of any targeted agent will be measured by whether or not it provides clinical benefit to patients. In this respect, the current design of clinical trials involving antiangiogenic agents may be improved by the development of rapid and effective biomarkers to establish drug dosage and monitor clinical response. Miller *et al* (2005) have recently reviewed the potential and limitations of a wide range of techniques used for imaging of tumour vasculature including positron emission tomography, X-ray computed tomography, magnetic resonance imaging, ultrasound and optical imaging. They also discussed which methods are likely to have the sensitivity and robustness for monitoring responses to cancer therapy and described ways in which imaging has been used in clinical trials to date. Such techniques will be important additional tools for assessing the clinical efficacy of the new drugs being developed.

The clinical utility of targeted agents is now established for both tumour and tumour blood vessel targets, with a number of agents already approved (Table 1) and others at various stages of clinical development. Nevertheless, challenges remain and several aspects of targeted therapy need to be optimised. These include treatment schedules and use in combination with certain conventional anticancer strategies or other targeted agents, as well as identification of those patients, disease settings and tumour types most likely to respond.

## Figures and Tables

**Table 1:** Monoclonal antibodies and tyrosine kinase inhibitors currently licensed for the treatment of cancer

**Drug**	**Molecular target(s)**	**Indication**
*Monoclonal antibodies*		
Bevacizumab (Avastin™)	VEGF	Colorectal cancer
Cetuximab (Erbitux™)	EGFR (HER-1)	Colorectal cancer
Trastuzumab (Herceptin®)	HER-2	Breast cancer
Rituximab (Rituxan®)	CD20	B-cell non-Hodgkin's lymphoma

*Tyrosine kinase inhibitors*		
Erlotinib (Tarceva™)	EGFR	Non-small-cell lung cancer
Gefitinib (Iressa™)	EGFR	Non-small-cell lung cancer
Imatinib (Glivec®)	Bcr-Abl PDGFR c-Kit	Chronic myeloid leukaemia Gastrointestinal stromal tumours

‘Iressa’ is a trademark of the AstraZeneca group of companies
